# Novel *Drexlerviridae* bacteriophage KMI8 with specific lytic activity against *Klebsiella michiganensis* and its biofilms

**DOI:** 10.1371/journal.pone.0257102

**Published:** 2021-09-07

**Authors:** Heng Ku, Mwila Kabwe, Hiu Tat Chan, Cassandra Stanton, Steve Petrovski, Steven Batinovic, Joseph Tucci

**Affiliations:** 1 Department of Pharmacy and Biomedical Science, La Trobe Institute for Molecular Science, La Trobe University, Victoria, Australia; 2 Department of Physiology, Anatomy and Microbiology, La Trobe University, Victoria, Australia; 3 Department of Microbiology, Royal Melbourne Hospital, Victoria, Australia; Universidade de Aveiro, PORTUGAL

## Abstract

The bacterial genus *Klebsiella* includes the closely related species *K*. *michiganensis*, *K*. *oxytoca* and *K*. *pneumoniae*, which are capable of causing severe disease in humans. In this report we describe the isolation, genomic and functional characterisation of the lytic bacteriophage KMI8 specific for *K*. *michiganensis*. KMI8 belongs to the family *Drexlerviridae*, and has a novel genome which shares very little homology (71.89% identity over a query cover of only 8%) with that of its closest related bacteriophages (Klebsiella bacteriophage LF20 (MW417503.1); Klebsiella bacteriophage 066039 (MW042802.1). KMI8, which possess a putative endosialidase (depolymerase) enzyme, was shown to be capable of degrading mono-biofilms of a strain of *K*. *michiganensis* that carried the polysaccharide capsule KL70 locus. This is the first report of a lytic bacteriophage for *K*. *michiganensis*, which is capable of breaking down a biofilm of this species.

## Introduction

The closely related *Klebsiella* bacteria *K*. *pneumoniae* and *K*. *oxytoca* form part of the normal human flora, but are also known to cause serious infections. *K*. *pneumoniae*, a member of the group of ESKAPE pathogens (*Enterococcus faecium*, *Staphylococcus aureus*, *Klebsiella pneumoniae*, *Acinetobacter baumannii*, *Pseudomonas aeruginosa* and *Enterobacter* spp), can cause pneumonia, urinary tract infections, and wound infections [1–3]. *K*. *oxytoca* is implicated in up to half of the cases of antibiotic associated haemorrhagic colitis and antibiotic associated diarrhoea [4, 5]. *K*. *oxytoca* has also been shown to contribute to the severe weight loss in cancer anorexia/cachexia, as its prevalence is increased, and it may alter gut barrier function, in this condition [6]. In 2013, a novel species of the genus *Klebsiella*, *K*. *michiganensis*, was first identified and characterised [7]. Its closest phylogenetic relative is *K*. *oxytoca*, with which it shares 99% nucleotide sequence identity in the 16S rRNA gene sequence [7]. Although there have only been limited studies on *K*. *michiganensis* to date, it is being recognised as an emerging pathogen [8]. It has been associated with sepsis in immunocompromised patients [9], and nosocomial infections [10], including in neonatal units [11] where it can cause necrotizing enterocolitis, a potentially fatal disease affecting the gut of premature infants [8]. In this connection, it has been suggested to be more clinically relevant to a subset of preterm infants than *K*. *oxytoca* [8]. Further, as *K*. *michiganensis* is often misidentified as *K*. *oxytoca*, despite clinical laboratories deploying the latest technology available to them [11], some of the conditions attributed to *K*. *oxytoca* may be caused by *K*. *michiganensis* instead. Similar to *K*. *oxytoca* and *K*. *pneumoniae*, *K*. *michiganensis* has been shown to carry a range of resistance mechanisms, including genomic and plasmid mediated extended spectrum β-lactamases [8, 11], carbapenemases [9, 10, 12], and multi drug resistance efflux pumps [10]. Some of these resistance factors may have been acquired through horizontal gene transfer with other enteric bacterial species [10].

Bacterial resistance to antibiotics, which was warned about by Alexander Fleming in his Nobel Prize speech in 1945 [13], is now one of the biggest problems facing medicine. Another factor which increases bacterial virulence is the capacity to form biofilms. While both *K*. *pneumoniae* and *K*. *oxytoca* are able to form microbial biofilms [14–19], the capacity of *K*. *michiganensis* to do so has not been studied. The treatment of bacteria in these communities is hindered by the fact that antibiotics do not readily penetrate biofilms [20].

Bacteriophages may offer the hope of adjunct therapy for infectious diseases, and provide options in such treatment. These bacterial viruses are specific in their actions. They offer a useful tool in the selective manipulation of the microbiome, with minimal disruption to the non-targeted microflora, in a manner that antibiotics cannot achieve. They are also able to disrupt bacterial biofilms and kill antibiotic resistant bacteria [21–24]. In the past decade or so, medical research in Western countries has begun to focus on the potential for application of bacteriophages, with successful administration in clinical scenarios having been reported [25–27]. In some instances, not only was the clinical response noted, but also total eradication of the pathogen, a scenario which is not seen with conventional antibiotic treatment [28]. That bacteriophages administered intravenously in the treatment of severe bacterial infections are safe and well tolerated, has also been reported [29].

We and others have previously reported the isolation and characterisation of bacteriophages lytic against *K*. *oxytoca* and *K*. *pneumoniae* [30–32]. While the host range of most bacteriophages is limited to strains within a single species, there have been reports of bacteriophages which are able to kill both *K*. *oxytoca* and *K*. *pneumoniae* [30, 31, 33]. This extended host range killing across species is most likely due to the very close homology between *K*. *oxytoca* and *K*. *pneumoniae*. *K*. *pneumoniae* associated capsules have been found among *K*. *oxytoca* isolates [34, 35], which may help to explain why some bacteriophages are able to lyse both strains. The initial report of the characterisation of *K*. *michiganensis* also described it as containing a capsule [7].

We report here the isolation, genomic and functional characterisation of KMI8, a novel lytic bacteriophage which is specific for *K*. *michiganensis*. KMI8 shares very little genetic similarity with other *Klebsiella* bacteriophages, and is shown here to be capable of degrading *K*. *michiganensis* biofilms *in-vitro*. This is the first report of a bacteriophage specific for *K*. *michiganensis* and which is capable of degrading its biofilms. The potential exists for further testing of bacteriophages such as KMI8 in the treatment of biofilms and infections of this bacteria.

## Methods

### Ethics approval

All methods were performed in accordance with the La Trobe University Ethics, Biosafety and Integrity guidelines and regulations. Clinical isolates of *K*. *michiganensis*, *K*. *oxytoca* and *K*. *pneumoniae* were obtained from specimen cultures as part of routine care. All samples were obtained with oral consent, de-identified and handled according to the La Trobe University Ethics committee. No ethical concerns were raised as human material was not the focus of the study and bacteria obtained were not traced back to the individual. The study protocols were approved by the La Trobe University Ethics Committee, reference number: S17-111.

### Bacterial cultures and identification

The 11 *Klebsiella* strains used here (Table 1) were grown using tryptone soy broth (TSB) and 1.2% agar (Oxoid, Adelaide, Australia), incubated at 37ºC aerobically for two days. Preliminary identification of the strains was performed by matrix assisted laser desorption ionisation-time of flight mass spectrometry (MALDI-TOF, Bruker Daltonik, Germany), and 16S rRNA gene sequencing. For extraction of bacterial DNA, a loopful of colonies was resuspended in 50 μL of nuclease free water in a 1.5 mL microcentrifuge tube and briefly vortexed. To this was added 2 μL of 0.5 M ethylenediaminetetraacetic acid (EDTA) (pH 8.0), 2.5 μL of 10% (w/v) sodium dodecyl sulphate (SDS) and 2.5 μL of proteinase K (20 mg/mL). The contents were gently mixed by inverting and incubated at 55 °C for one h. After cooling to room temperature, 150 μL of nuclease free water and 200 μL of phenol: chloroform: isoamyl alcohol (29:28:1) were added and mixed by inverting 10 times and vortexed until cloudy. The cloudy suspension was centrifuged at 12000 x *g* for 10 min. The clear upper aqueous layer was pipetted into a fresh 1.5 mL microcentrifuge tube containing an equal volume of isopropanol and incubated overnight at -20 °C. The tube was then centrifuged for 10 min at 12000 x *g*. The supernatant was discarded and 200 μL of 70% ethanol added to the DNA pellet. This was centrifuged for 5 min at 12000 x *g*, then the ethanol discarded. The DNA pellet was air dried before resuspending in 50 μL of nuclease free water. PCR amplification of the 16S rRNA gene of each bacterial strain was performed using universal primers 27F (5’-AGAGTTTGATCMTGGCTCAG-3’), 519R (5’-GWATTACCGCGGCKGCTG-3’), 895F (5’-RCCTGGGGAGTRCRG-3) and 1492R (5’-GGTTACCTTGTTACGACTT-3’) [32, 36–38]. Go Taq^®^ Long PCR Master Mix (Promega, Sydney, Australia) was used for the PCR amplification. The samples were subjected to an initial denaturation step of 2 min at 95°C, followed by 30 amplification cycles (94°C, 20 s; 59°C, 20 s; 72°C, 30 s) and a final elongation step of 10 min at 72°C. PCR products were analysed by electrophoresis in 1.2% agarose gels, stained with ethidium bromide and visualised under ultraviolet light [39]. Products were cleaned with an Ultra Clean^®^ DNA purification kit (MO BIO San Diego), before submission to the Australian Genome Research Facility for Sanger sequencing.

**Table 1:** *Klebsiella spp*. used for KMI8 isolation and host range studies.

Lab name and genbank accession number (in parenthesis)	MALDI-TOF identification	Closest BLASTn genbank match (16s rRNA sequence)	Taxonomy based on WGS
KLEB001 (JAERQC000000000)	*K*. *pneumoniae*	*K*. *pneumoniae* strain DSM30104	*K*. *pneumoniae*
KLEB002 (JAERQB000000000)	*K*. *pneumoniae*	*K*. *pneumoniae* strain DSM30104	*K*. *pneumoniae*
KLEB006 (JAERQA000000000)	*K*. *pneumoniae*	*K*. *pneumoniae* strain DSM30104	*K*. *pneumoniae*
KLEB007 (JAERPZ000000000)	*K*. *pneumoniae*	*K*. *pneumoniae* strain DSM30104	*K*. *pneumoniae*
KLEB009 (JAERPY000000000)	*K*. *pneumoniae*	*K*. *pneumoniae* strain DSM30104	*K*. *pneumoniae*
KLEB011 (JAEQMJ000000000)	*K*. *oxytoca*	*K*. *oxytoca* strain JCM1665	*K*. *michiganensis*
KLEB012 (JAERPX000000000)	*K*. *oxytoca*	*K*. *oxytoca* strain NBRC 102593	*K*. *oxytoca*
KLEB013 (JAERPW000000000)	*K*. *oxytoca*	*K*. *oxytoca* strain JCM1665	*K*. *michiganensis*
KLEB014 (JAERPV000000000)	*K*. *oxytoca*	*K*. *oxytoca* strain JCM1665	*K*. *michiganensis*
KLEB015 (JAERPU000000000)	*K*. *oxytoca*	*K*. *oxytoca* strain JCM1665	*K*. *michiganensis*
KLEB016 (JAERPT000000000)	*K*. *oxytoca*	*K*. *oxytoca* strain JCM1665	*K*. *michiganensis*

The 11 *Klebsiella* strains were then definitively identified by whole genome sequencing (WGS). DNA extracted from the *Klebsiella* bacterial strains was sequenced using an Illumina MiSeq^®^ next generation sequencing platform. The DNA library was prepared by using the NEBNext^®^ Ultra^™^ II DNA Library Prep Kit according to the manufacturer’s instructions and sequenced using a MiSeq^®^ V2 300-cycle reagent or V3 600-cycle kit to generate 150 bp or 300 bp paired end reads. Short-read data were filtered using Trim Galore v0.6.4 with the default settings (Q scores of ≥20, with automatic adapter detection). Reads were assembled *de novo* using Unicycler v0.4.8. Open reading frames of these sequences were predicted using Prokka 1.14.5. Bacterial taxonomy was assessed based on average nucleotide identity (ANI) using JSpeciesW [40] and the GTDBK-Tk software toolkit for assigning objective taxonomic classifications to prokaryotic genomes. These results were confirmed by ANI analysis results from NCBI. The program Staramr V.0.7.2 (Galaxy Australia) and the databases ResFinder, PlasmidFinder, and PointFinder were used to identify antibiotic resistance genes in the genomes of KLEB011, KLEB014 & KLEB015. We employed the program CRISPRcasFinder to identify any potential CRISPR regions in the genomes of the *Klebsiella* strains used here. The *K*. *michiganensis* KLEB011 genome was analysed using the Kaptive Web online tool for the rapid typing of *Klebsiella* K (polysaccharide capsule) and O (lipopolysaccharide) loci [41].

### Isolation and purification of bacteriophages

Wastewater from Victoria, Australia, was used to screen for bacteriophages. Briefly, bacterial lawns of *K*. *michiganensis*, *K*. *pneumoniae* and *K*. *oxytoca* on Tryptone soy agar (TSA) (Oxoid, Australia) were prepared by using a cotton swab to spread onto the 1.2% agar plates a sample from a log phase culture. Bacteria and wastewater samples were incubated for 2 d in TSB (Oxoid, Adelaide, Australia) at 37 ºC, then centrifuged (12,000 × *g* for 5 min) and filtered through 0.2 μM pore cellulose acetate filters (Advantec, Melbourne, Australia). The filtrate (10 μL) was placed onto the bacterial lawn and plates were incubated for 24 h. Any potential bacteriophage clearing was excised (along with a portion of agar) and resuspended in 500 μL of TSB in a 1.5 mL microcentrifuge tube, before centrifugation (12,000× *g* for 5 min) and a 10-fold serial dilution was completed. A total of 10 μL of each dilution was placed on a bacterial lawn to observe plaques. This serial dilution purification was repeated five times to ensure single virion infection. After isolation and purification of bacteriophage, an enrichment method was used to prepare high concentration phage stocks. 300μL of bacteriophage solution was incubated with 500μL of bacteria (log phase) in a final volume of 10mL TSB at 37°C. This process was repeated several times using the previous concentrated bacteriophage stocks until the bacteriophage concentration of >10^7^ PFU/mL was reached.

### Extraction of bacteriophage DNA

Five mL of bacteriophage stock (>10^9^ PFU/mL) was used to extract bacteriophage DNA. All chemicals were obtained from Sigma-Aldrich (Sydney, Australia) unless stated otherwise. The bacteriophage stock was digested at room temperature for 30 min in 5 mmol/L MgCl_2_, 10 μg/mL DNase I and RNase A to remove any bacterial nucleic acids present. The bacteriophages were recovered by overnight polyethylene glycol (PEG) precipitation using 10% (w/v) PEG 8000 and 1 mol/L NaCl at 4°C. Precipitated bacteriophages were centrifuged (12,000× *g*) for 15 min and resuspended in nuclease free water (Promega, Sydney, Australia). Bacteriophage proteins were digested for one h at 55°C by adding 50 μg/mL Proteinase K, 20 mmol/L EDTA and 0.5% (*v*/*v*) SDS. Following this, an equal volume of phenol–chloroform–isoamyl alcohol (29:28:1) was added and centrifuged (12,000× *g*) for 5 min. The aqueous phase was removed and an equal volume of isopropanol was added to it. The DNA was precipitated overnight at −20°C, then collected by centrifugation (12,000× *g*) for 10 min. The DNA pellet was washed with 70% ethanol, air dried and resuspended in 25 μL of nuclease free water (Promega).

### Host range analysis

To assess for lytic capacity, bacteriophage stocks (>10^7^ PFU/mL) were diluted 10-fold serially and tested on *K*. *michiganensis*, *K*. *oxytoca* and *K*. *pneumoniae* strains. A bacterial lawn of the strain was prepared and 10 μL of each dilution of the stock was placed on the lawn. If individual plaques were observed, it was noted that the bacteriophage could target the strain. The efficiency of plating was calculated as the PFU/ml of the bacteriophage on each strain divided by, and relative to, the PFU/ml obtained on the strain on which it most efficiently lysed, multiplied by 100.

### Transmission electron microscopy

Transmission electron microscopy (TEM) was performed using 400-mesh formvar and carbon copper grids (ProSciTech, Townsville, Australia). Bacteriophage stocks (> 10^7^ PFU/ mL) were allowed to adsorb to the grid for one min before excess solution was removed using filter paper. The grids were then negatively stained three times with 2% (w/v) uranyl acetate for 20 s. Excess stain was removed by filter paper and grids were air-dried for 20 min before examination under a JEOL JEM-2100 transmission electron microscope. This was operated at an accelerating voltage of 200 kV and high-resolution digital images were recorded on a Gatan Orius SC200D 1 wide angle camera with Gatan Microscopy Suite and Digital Micrograph (Version 2.32.888.0) imaging software. Virions were measured using ImageJ software [42] (Version 1.8.0_112).

### Bacteriophage genomic characterisation and phylogeny

DNA extracted from the bacteriophage was sequenced using an Illumina MiSeq^®^ next generation sequencing platform. The DNA library was prepared by using a Nextera^®^ XT DNA sample preparation kit according to the manufacturer’s instructions and sequenced using a MiSeq^®^ V2 reagent kit (300 cycles), as 150 bp paired end reads. Short-read data were filtered using Trim Galore v0.6.4 with the default settings (Q scores of ≥20, with automatic adapter detection). Reads were assembled de novo within Unicycler v0.4.8. Open reading frames of these sequences were predicted using Glimmer, Prodigal V.2.6.2 and manually checked by Geneious prime V.2019.0.3. The bacteriophage genome was annotated using Blastp (Version 2.9.0) nr database with a cut off e-value of (1e-5), NCBI Web CD-search Tool (Pfam database) with a cut off e-value of 0.01 and HHpred (default database:PDB_mmCIF70_23_Jul; Pfam-A_V33.1; NCBI_Conserved Domain (CD)_v3.18). The proteomic tree was constructed with ViPTreeGen (Version 1.1.2) using sequence similarity distance based on tBLASTx results and construct (bio)nj tree. EvolView was used to annotate the proteomic tree. tRNAs were predicted by ARAGORN and tRNAscan-SE-2.0.5.

### One step growth analysis

The one step growth analysis was performed on three replicates as described previously [43]. *K*. *michiganensis* strain KLEB011 was grown to exponential phase in TSB, and harvested by centrifugation at 12,000× g for 5 min. 1x10^6^ bacterial cells were resuspended in fresh 900 μL TSB to which 100 μL of KMI8 bacteriophage stock was added at a multiplicity of infection (MOI) of 1, and incubated at 4°C for 5 min to allow for adsorption. Adsorbed bacteriophages were collected by centrifugation at 12,000× g for 5 mins and resuspended in 50 mL of fresh TSB. The mixture was incubated aerobically at 37°C. One mL aliquots were taken every 10 min and bacteriophage concentration (PFU/mL) assayed by centrifugation at 12,000× *g* for 2 min before 10-fold serial dilution and placing 10 μL of these dilutions on a fresh lawn of *K*. *michiganensis* strain KLEB11. These were incubated aerobically overnight to observe bacteriophage plaque formation.

### Biofilm growth and quantification

The biofilm experiment was conducted aerobically using TSA broth (Oxoid, Scoresby, Australia) supplemented with 0.5% glucose in 96 well polystyrene plates (Greiner bio-one, Frickenhausen, Germany) coated with 0.5% gelatin (Sigma-Aldrich, Sydney, Australia). The *K*. *michiganensis* strain KLEB11 was cultured overnight, and 100 μL of 10^8^ CFU/mL of this bacteria in exponential growth phase was added to each well with an equivalent volume of fresh TSA broth. The 96 well plates were incubated shaking at 37°C under aerobic conditions for 3 d with sterile broth replenishment every 24 h. After the biofilm was formed, bacteria in suspension were removed before 100 μL of bacteriophage KMI8 at 10^8^ PFU/mL was added to each well and incubated for a further 2.5 h, before quantification assays completed as described previously [44]. Briefly, unattached *K*. *michiganensis* cells were washed off by submerging in Milli-Q^®^ deionised water (Merck, Bayswater, Australia) for 5 min and air dried before staining with 200 μL of 0.1% (v/v) crystal violet for 10 min. Excess crystal violet stain was washed off twice by resuspension in Milli-Q^®^ deionised water (Merck, Bayswater, Australia) for another 5 min before leaching out the cell bound crystal violet in 70% (v/v) ethanol. Absorbance in each well was determined using a FlexStation 3 plate reader (Molecular Devices, USA) at a wavelength of 600 nm.

### Biofilm viability analysis

Biofilm viability was determined as previously described [44]. Briefly, the *K*. *michiganensis* biofilm was grown on gelatin-coated microscope coverslips and treated with bacteriophage KMI8 in the same manner as the 96 well plates described above. SYBR^®^ gold and propidium iodide (PI) were used to stain nucleic acids of all cells (including live membrane intact and dead, membrane compromised cells). 2 μL of a 1 mg/mL concentration of PI in dimethyl sulfoxide (Sigma-Aldrich, Australia) was added to 0.2 μL of a 1 mg/mL concentration of SYBR^®^ gold and resuspended in 100 μL of nuclease free water (Promega, Australia). The mixture was applied to the biofilm on slides and incubated for 15 min in the dark. Excess stain was then washed off in nuclease free water (Promega, Sydney, Australia) and the coverslips mounted on microscope slides using vectorshield^®^ (Burlingame, CA, USA). Slides were immediately examined under the Fluoview Fv10i-Confocal laser-scanning microscope (Olympus Life Science, Notting Hill, Australia) at excitation wavelength of 485 nm and emissions at 535 nm and 635 nm for green and red fluorescence, respectively. SYBR^®^ gold stains all cells green while PI stains all membrane compromised cells red. When these stains were combined and cells imaged as in these experiments, cells which were stained green indicated live cells, and those not green, (red or orange) indicated membrane compromised dead cells. A quantitative analysis of the viable bacterial cells, with and without bacteriophage treatment, was performed. For these analyses, we scanned and counted cells in 14 different areas of the coverslips for bacteriophage treated and untreated biofilms, across triplicate experiments.

### Statistical analysis

The absorbance values quantifying the biofilms were analysed for normality using the Shapiro Wilk test and since all data were normal, their means compared by a paired T-test. For the biofilm viability examination using confocal microscopy, a Shapiro-Wilk test showed that some data were normally distributed while some were not normally distributed. As such, a non-parametric test (2-independent samples test), the Mann-Whitney U test, was used to analyse the data. A p-value of less than 0.05 was considered statistically significant. All statistical analysis was performed using the Statistical Package for Social Sciences (SPSS version 25).

### Codon usage analysis

Geneious prime V.2020.0.3 was employed to extract nucleotide sequences from genome annotations of the *K*. *michiganensis* strain KLEB011 and KMI8. Biopython script was written and used to remove the start codons by slicing nucleotide sequence [45, 46]. *K*. *michiganensis* KLEB011 and KMI8 codon usage was calculated using python script and findings confirmed with the Sequence Manipulation Suite of JavaScript programs for analyzing and formatting protein and DNA sequences [47].

Codon usage analyses to investigate the adaptive role of bacteriophage encoded tRNAs have been described previously [48]. Briefly, for the 64 codons, a relative codon frequency, *fi*, was calculated using the following formula: *f*_*i*_ = *Number of codon*_*i*_*/ Number of all codons*. The ratio of relative codon frequencies, *ri*, between bacteriophage and host was then assessed as: *r*_*i*_ = *f*_*i*_^*bacteriophage*^*/f*_*i*_^*host*^. If *ri* was ≥ 1.1, the bacteriophage was considered to exhibit a higher relative codon frequency for codon *i* than the host; If *ri* was between 0.9 and 1.1, the bacteriophage and host were considered to have similar relative codon frequencies; if *ri* was < 0.9, the host was considered to exhibit a higher relative codon frequency [48].

## Results

### Definitive identification of *Klebsiella* strains for use in KMI8 isolation

*Klebsiella* bacterial strains used in this study were identified and differentiated using a combination of MALDI-TOF, 16S rRNA gene sequencing, and WGS (Table 1). The use of MALDI-TOF and 16S rRNA gene sequencing were successful in differentiating *K*. *pneumoniae* from the other species used in this study (Table 1). However, they were unsuccessful in differentiating *K*. *oxytoca* from *K*. *michiganensis*: these techniques incorrectly identified each of KLEB11-KLEB16 as *K*. *oxytoca*. In contrast, WGS analysis identified only KLEB12 as *K*. *oxytoca*, while KLEB11 and KLEB13-KLEB16 were identified as *K*. *michiganensis* (Table 1). Using a program employed by NCBI, JSpeciesWs, we identified the % genomic similarity of the strains used here. The program uses Average Nucleotide Identity (ANI) calculations based on BLAST+ to calculate the % genomic similarity between these strains. S1 Table shows the average nucleotide identity between the *Klebsiella* strains used in this study.

### Identification of antibiotic resistance genes in the genomes of KLEB011, KLEB014 & KLEB015, the *K*. *michiganensis* strains lysed by KMI8

The program Staramr V.0.7.2 (Galaxy Australia) and databases ResFinder, PlasmidFinder, and PointFinder were used to identify antibiotic resistance genes in the genomes of KLEB011, KLEB014 & KLEB015 (Table 2).

**Table 2:** Antibiotic resistance genes in *K*. *michiganensis* strains KLEB011, KLEB014 & KLEB015.

Strain ID	Genotype	Predicted resistance phenotype
KLEB011	blaOXY-1-4	ampicillin
KLEB014	aph(3’)-Ia, blaOXY-1-7	kanamycin, ampicillin
KLEB015	aph(3’)-Ia, blaOXY-1-3	kanamycin, ampicillin

### Capsule locus identification for *K*. *michiganensis* strain KLEB011 used in one step growth curve and biofilm assays

Use of the Kaptive Web tool for the rapid typing of *Klebsiella* K (polysaccharide capsule) and O (lipopolysaccharide) loci [41] revealed that the KLEB011 genome carried the capsule KL70 locus.

### Isolation, host range analysis, and phenotypic characterization of novel *K*. *michiganensis* bacteriophage KMI8

KMI8, a novel bacteriophage isolated from samples of wastewater from Victoria, Australia, was capable of lysing three *K*. *michiganensis* strains (KLEB011, KLEB014 & KLEB015) but did not kill any of the *K*. *oxytoca* or *K*. *pneumoniae* strains tested. We employed the program CRISPRcasFinder to identify any potential CRISPR regions in the genomes of the *Klebsiella* strains used here. These genomes appear to be devoid of any functional anti-KMI8 CRISPR or any evidence of sufficient Cas genes for a functional CRISPR-Cas system. The efficiency of plating for KMI8 on its host *K*. *michiganensis* strains was KLEB011: 100%; KLEB014: 3.2%; KLEB015: 0.48% (calculated as the PFU/ml of KMI8 on each strain divided by, and relative to, the PFU/ml obtained on strain KLEB011, on which it most efficiently lysed, multiplied by 100).

**Fig 1:**
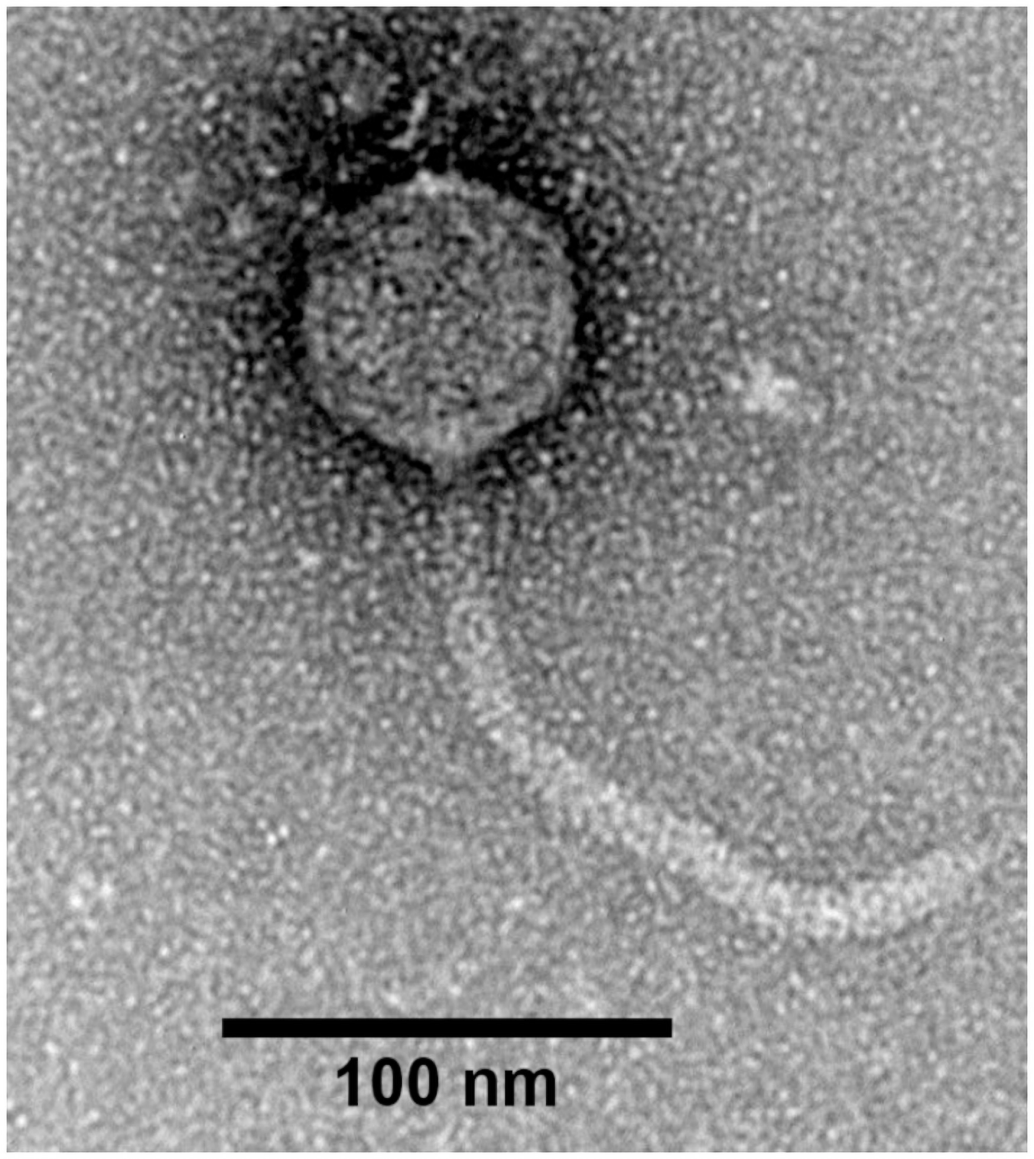
TEM characterisation of bacteriophage KMI8: Siphovirus with capsid length of 71nm ± 2nm; tail length and width approximately 190 nm ± 5 nm and 10 ± 0.1 nm, respectively.

**Fig 2:**
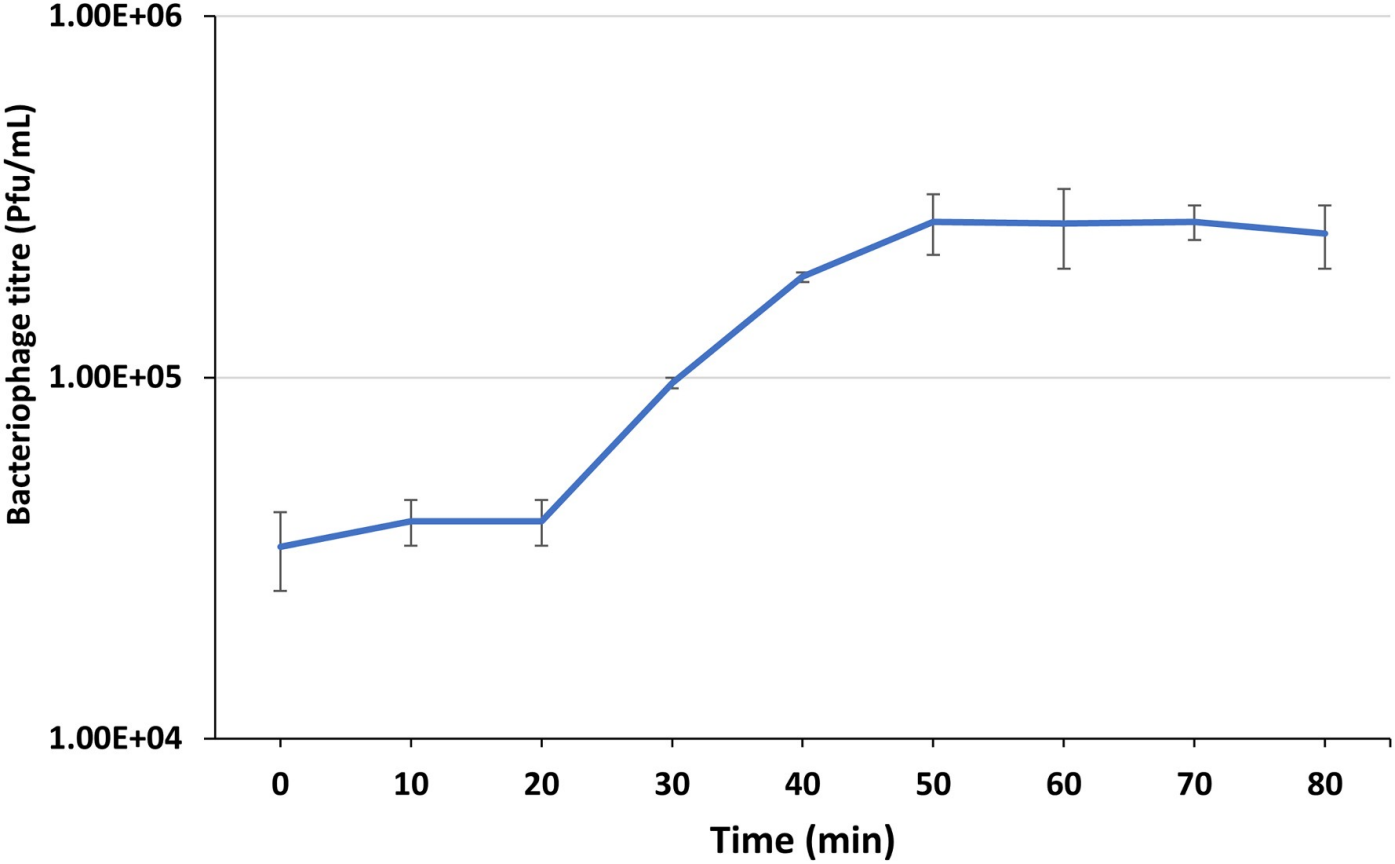
KMI8 one step growth curve. Error bars represent standard error of mean, calculated from three independent experiments.

Plaques formed by KMI8 on 1.2% agar were approximately 1 mm in diameter. TEM revealed that KMI8 was of the siphovirus morphotype (Fig 1). KMI8 had a capsid length of approximately 71 nm ± 2 nm; tail length and width approximately 190 nm ± 5 nm and 10 ± 0.1 nm, respectively. In a study of the growth kinetics of KMI8 grown on KLEB11, one step growth analysis showed latent and burst periods of 20 min and 30 min respectively (Fig 2), and a burst size of 12 ± 2 PFU/infected bacteria.

### Genome analysis of *K*. *michiganensis* bacteriophage KMI8

**Fig 3:**
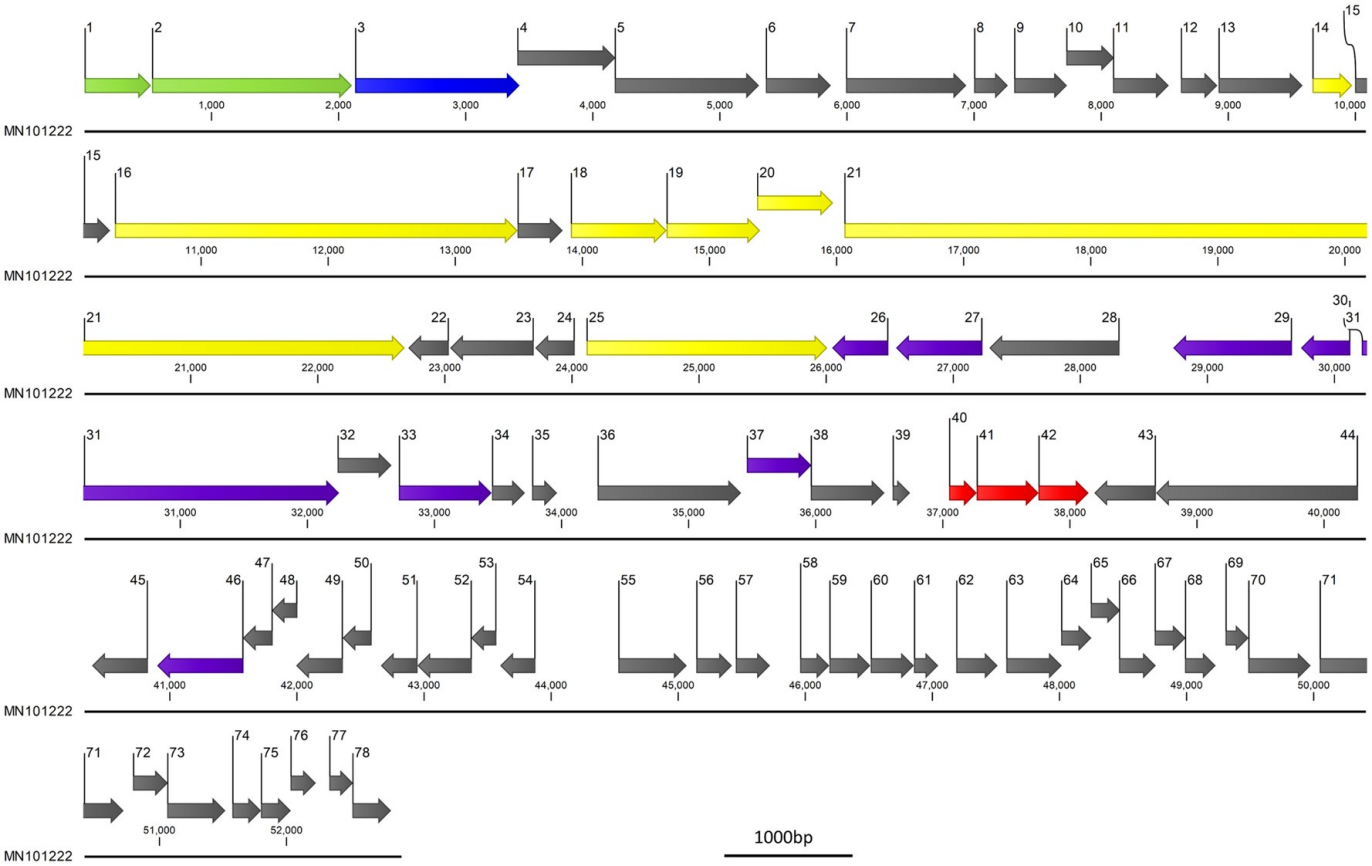
KMI8 (MN101222) genome. Arrows indicate putative ORFs (numbered) and direction of transcription. Grey arrows indicate putative hypothetical genes; green arrows indicate putative terminase genes; blue arrow indicates putative capsid gene; yellow arrows indicate putative tail fibre genes; purple arrows indicate putative DNA manipulation and metabolism genes; red arrows indicate putative lysis genes. Scale bar = 1000bp.

The genome sequence data was assembled *de novo*. The KMI8 bacteriophage genome (Accession number: MN101222) had a genome size of 52904 bp (with an average coverage of 474x) and was composed of 78 predicted open reading frames (ORFs) (Table 3 and Fig 3) of which 26.9% (21/78) were related to genes encoding putative functional proteins (Table 4). The GC content of KMI8 was 49.3% (Table 3) (the GC content of one of its hosts, the *K*. *michiganensis* strain KLEB11, was 55.7%) and the closest related organism is *Klebsiella* bacteriophage 066039 (MW042802) with 71.89% identity over a query cover of only 8%. No putative integrase genes, toxin genes, tRNA or Transfer-messenger RNA (tmRNA) genes were detected in the genome of KMI8. We assessed the bacteria it lyses, and did not detect the KMI8 genome in the genome of its host bacteria, nor in extracts of bacterial cells.

**Table 3:** KMI8 genome data and closest match.

Genome Size	GC content	Accession Number	Closest Related Organism; (Similarity)
52904 bp	49.3%	MN101222	*Klebsiella* bacteriophage 066039, accession: MW042802.1; (71.89% identity; query cover 8%)

**Table 4:** KMI8 ORFs where predicted protein function is known. Predicted function based on Pfam database of conserved functional domains.

ORF number	Predicted protein size (aa)	E-Value	Accession	Definition
1	173	4.06E-07	pfam16677	DNA-packaging protein gp3
2	712	9.23E-09	pfam17289	Terminase RNaseH-like domain
4	256	0.003087	pfam04233	Phage Mu protein F like protein
13	219	5.93E-54	pfam08813	Phage tail tube protein, TTP
14	103	1.92E-34	pfam08748	Phage tail assembly chaperone
16	1055	1.15E-51	pfam06791	Prophage tail length tape measure protein
17	117	4.69E-21	pfam05939	Phage minor tail protein
18	250	1.03E-44	pfam05100	Phage minor tail protein L
19	244	4.16E-15	pfam00877	NlpC/P60 family
20	198	4.08E-12	pfam06805	Bacteriophage lambda tail assembly protein I
21	2206	4.55E-12	pfam13550	Putative bacteriophage tail protein
25	630	6.84E-08	pfam13884	Chaperone of endosialidase (embedded in putative tail fibre ORF)
26	146	6.81E-17	pfam00436	Single-strand binding protein family
27	225	1.78E-23	pfam04404	ERF superfamily
29	310	4.83E-06	pfam08273	Zinc-binding domain of primase-helicase
31	677	6.24E-06	pfam00271	Helicase conserved C-terminal domain
32	140	2.37E-09	pfam08774	VRR-NUC domain
33	242	1.62E-65	pfam05869	DNA N-6-adenine-methyltransferase (Dam)
37	168	0.000149	pfam03767	HAD superfamily, subfamily IIIB (acid phosphatase)
38	192	0.000193	pfam13238	AAA domain
41	161	3.31E-16	pfam00959	Phage lysozyme

### Disruption of *K*. *michiganensis* biofilm by bacteriophage KMI8

**Fig 4:**
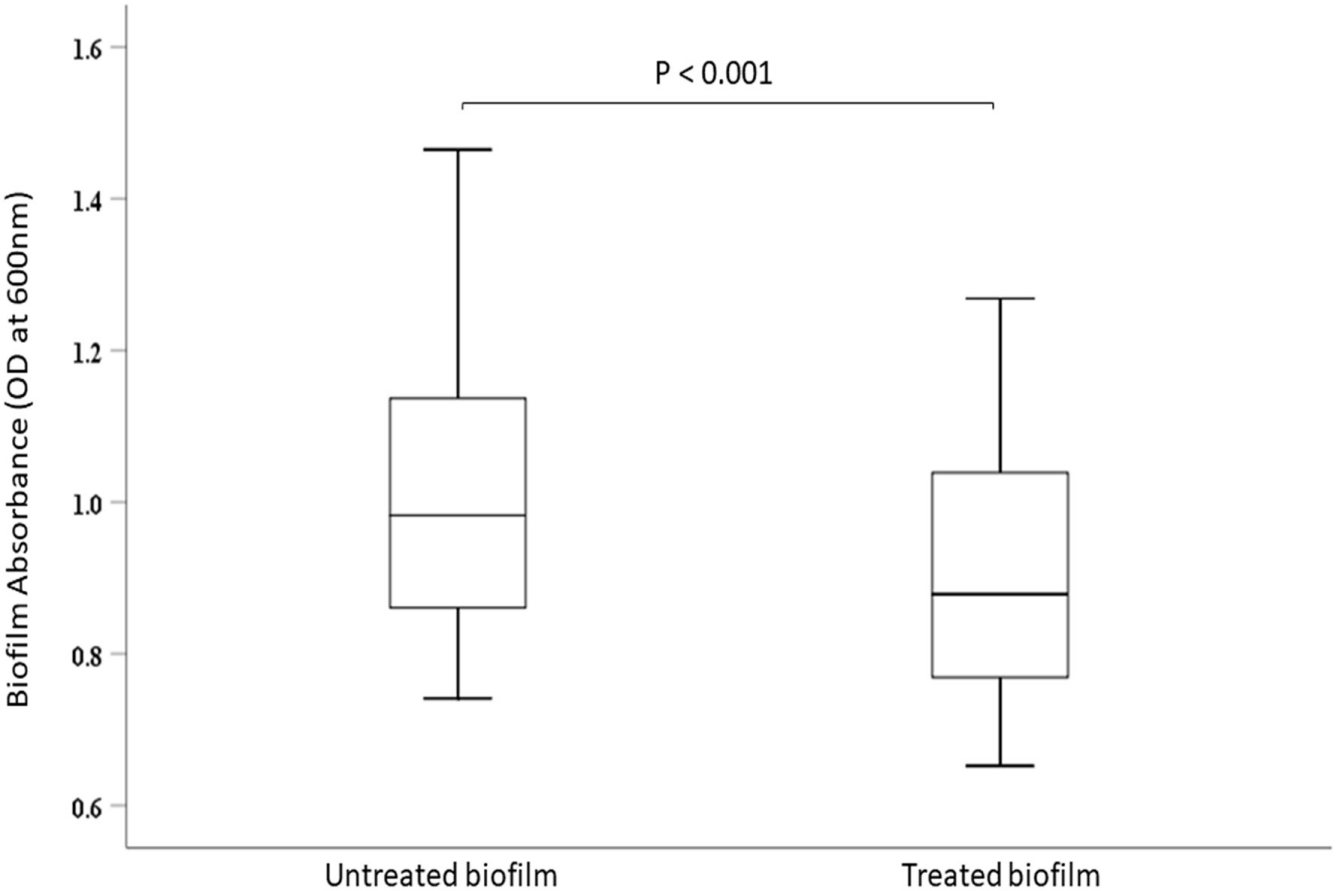
*K*. *michiganensis* biofilm absorbance measurements, showing significant reduction of *K*. *michiganensis* biofilm treated with KMI8 bacteriophage (p < 0.001).

**Fig 5:**
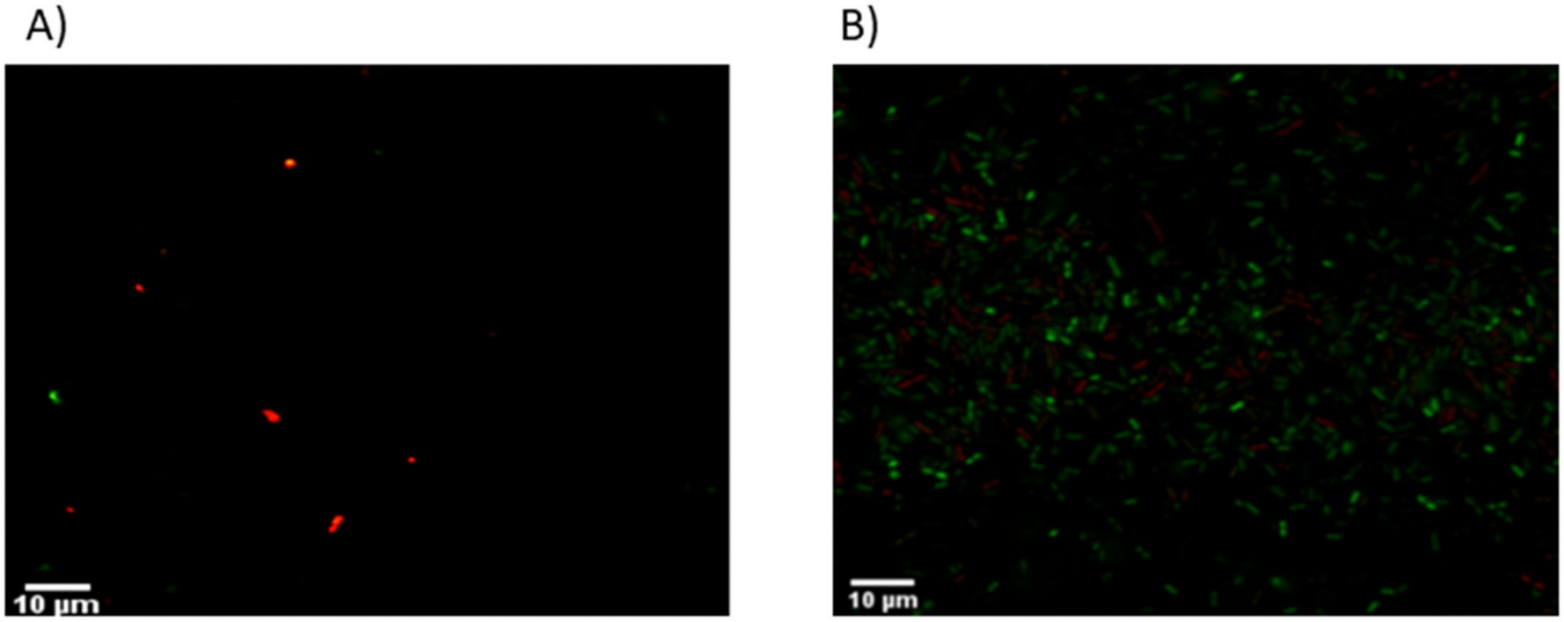
Confocal images of SYBR^®^ gold and PI staining following (A) bacteriophage treated and (B) untreated *K*. *michiganensis* biofilm. Green color indicates live cells, red/orange color indicates dead cells on the slides.

**Fig 6:**
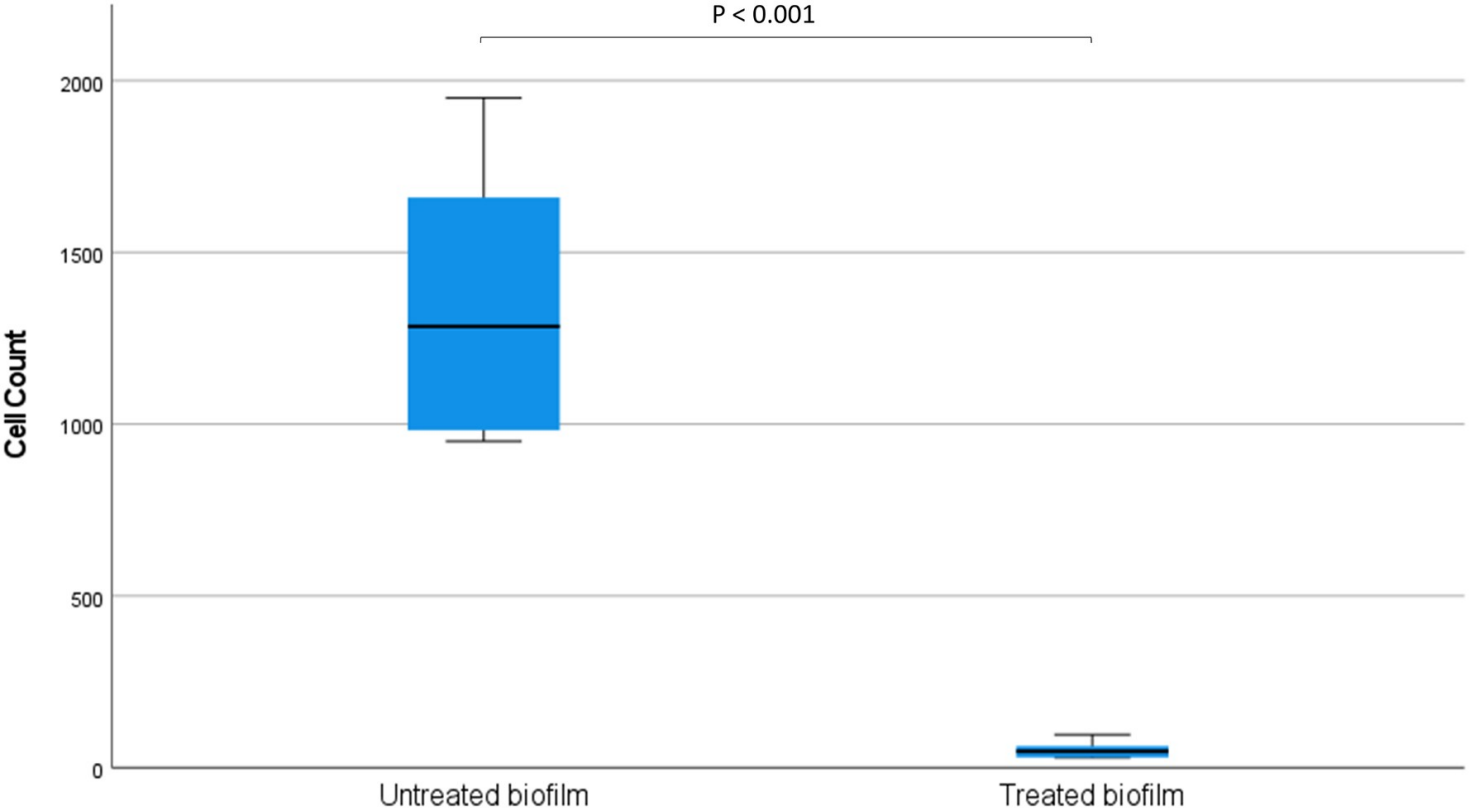
Viable cells in *K*. *michiganensis* biofilms before and after KMI8 treatment. There was a significant difference between untreated and treated biofilms (P < 0.001) (Mann-Whitney U test).

Bacteriophage KMI8 significantly lowered the *K*. *michiganensis* KLEB11 biofilm mass after 2.5 h exposure (p<0.001) (Fig 4). Confocal microscope imaging was used to visualise biofilm mass on glass slides. Control *K*. *michiganensis* biofilm mass (Fig 5B) and biofilm exposed to KMI8 for 2.5 h (Fig 5A) were stained with SYBR^®^ Gold and PI to indicate membrane intact live cells (green colour) and membrane compromised dead cells (red/orange colour). Biofilm mass showed many live cells attached to the glass slide in the untreated control (Fig 5B) while there was a sparse population of membrane compromised/dead bacteria in the KMI8 treated biofilm (Fig 5A). Counting of the viable cells in confocal images of *K*. *michiganensis* biofilms before and after KMI8 treatment revealed that there was a significant difference between untreated and treated biofilms (P < 0.001) (Mann-Whitney U test) (Fig 6).

### Bacteriophage phylogeny

**Fig 7:**
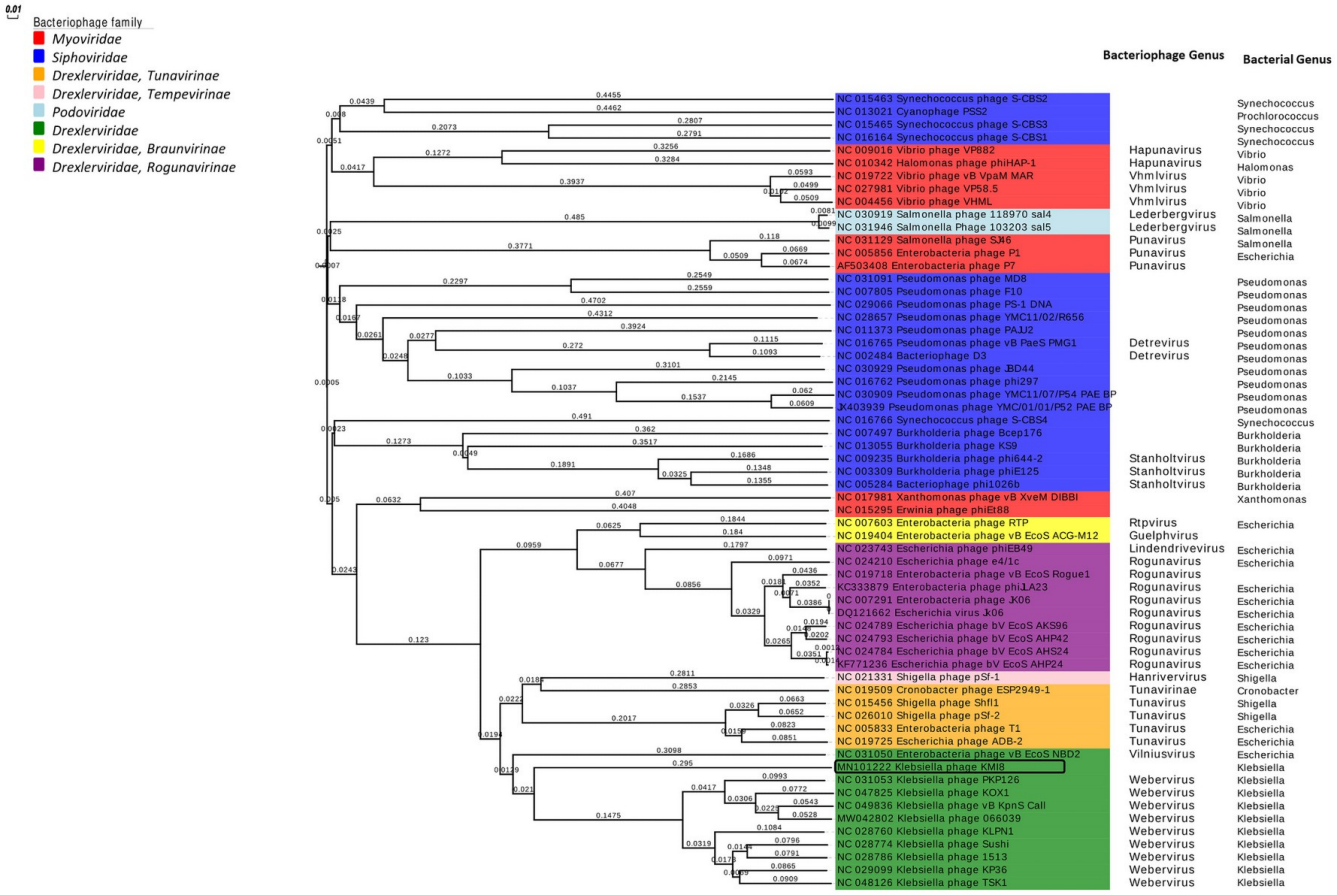
Proteomic tree showing the genome-wide proteomic diversity of KMI8 and related bacteriophages. Black box indicates KMI8.

ViPTree online was used to generate a proteomic tree based on genome wide sequence similarities, computed by tBLASTx, for KMI8 and 59 related bacteriophages. KMI8, which belongs to the family *Drexlerviridae*, is part of a monophyletic clade with other *Klebsiella* bacteriophages of the family *Drexlerviridae*, genus *Weberviridae* (Fig 7). It is unclear, however, to which genus KMI8 can be classified at this stage.

### Codon usage analysis in KMI8 and *K*. *michiganensis* strain KLEB11

**Fig 8:**
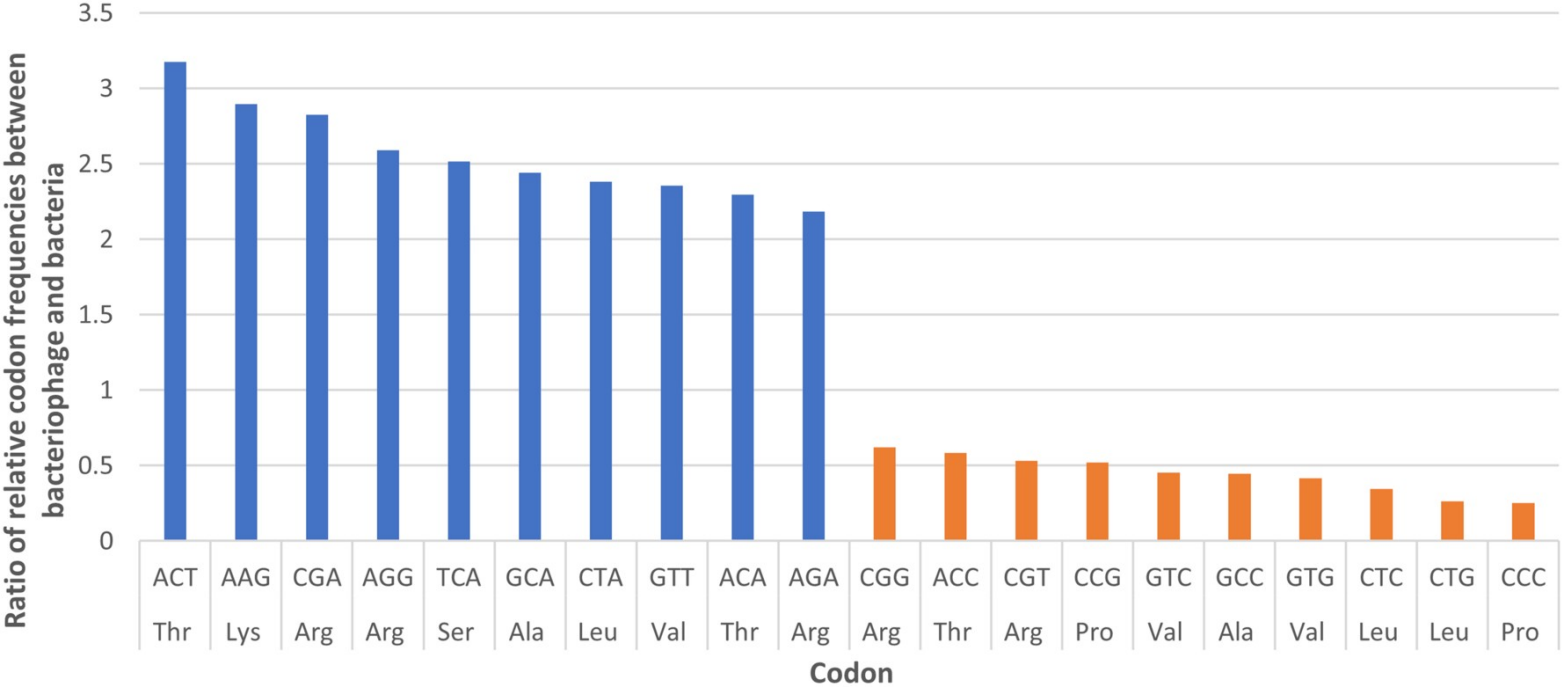
The ratio of relative codon frequencies between bacteriophage KMI8 and *K*. *michiganensis* strain KLEB011. The figure shows codons for which KMI8 exhibited the 10 highest (blue) and 10 lowest (orange) relative codon frequencies compared to the host.

Analysis of the relative codon frequencies for KMI8 and *K*. *michiganensis* strain KLEB11 revealed that the frequency of usage of five codons was at least two and a half times higher in KMI8, compared to the host *K*. *michiganensis* strain KLEB11 (Fig 8). The ratio of usage of ACT (threonine) by KMI8, compared to the host bacteria, was over three times greater.

## Discussion

We describe here a novel lytic bacteriophage, KMI8, which has very little genetic similarity to other reported bacteriophages. Compared to its closest relative, the *Klebsiella* bacteriophage 066039 (accession MW042802), its genome has 71.89% identity over a query coverage of only 8%. KMI8 appears to be specific for *K*. *michiganensis*. It is capable of lysing several strains of this bacteria, which carry resistance genes for ampicillin and kanamycin, but its host range does not extend to the *K*. *pneumoniae* or *K*. *oxytoca* strains tested here. In connection to this, our study showed that techniques such as MALDI-TOF and 16S rRNA gene sequencing were not sufficient to differentiate between *K*. *oxytoca* and *K*. *michiganensis*, and as shown here and by others, the precise definition of the phylogeny of these *Klebsiella* species requires more extensive applications such as WGS [8, 10, 11]. This is the first report of a bacteriophage lytic for *K*. *michiganensis*. However, it is possible that if WGS were used to refine the taxonomic analysis of closely related hosts of previously reported *Klebsiella* bacteriophages, some of these hosts would be reclassified as *K*. *michiganensis*. Further, given the genetic similarity between *K*. *michiganensis* and *K*. *oxytoca*, it is possible that known *K*. *oxytoca* bacteriophages may also target *K*. *michiganensis*.

KMI8 has a siphovirus morphology, with a long flexible non-contractile tail. It is a member of the *Drexlerviridae* family of viruses, and forms a monophyletic group with other bacteriophages lytic for *Klebsiella*, and which are all *Drexlerviridae* viruses of the genus *Webervirus*. It is unclear, however, to which genus KMI8 can be classified at this stage. Analysis of the growth kinetics of KMI8 showed a long latent period (20 min), and a rise period of up to 30 min during which the virus was bursting from the host cell, followed by a plateau. The burst size was 12 ± 2 PFU/infected bacteria. This compares to latency of 30 min and burst of 113 PFU/infected bacteria for the most closely related bacteriophage whose growth kinetics have been reported, the *Drexlerviridae Webervirus* TSK1 (accession MH688453) [49], which lyses *K*. *pneumoniae*. The GC content of the KMI8 genome was 49.3%, compared to that of one of its hosts, the *K*. *michiganensis* strain KLEB11, which was 55.7%. The putative function of most of the genome of KMI8 is unknown. We were only able to ascribe functionality for 21 of the 78 open reading frames it contains, based on the Pfam database of conserved functional domains. Its genes whose functions were predicted included putative terminase genes, putative capsid genes, putative tail fibre genes, putative DNA manipulation and metabolism genes, and putative lysis genes.

Analysis of the relative codon frequencies for KMI8 and the *K*. *michiganensis* strain KLEB11 revealed that the frequency of usage of a range of codons was higher in the bacteriophage, compared to the host. Previous suggestions have been for an adaptive role of bacteriophage encoded tRNAs, in that they are selected in bacteriophages to compensate for differences in codon usage between the bacteriophage and the host [50]. However, even though the ratio of usage of codon ACT (threonine) by KMI8 compared to the host bacteria was over three times greater, KMI8 possessed no tRNA or tmRNA genes for the anticodon corresponding to this or other codons. The reasons for this are unclear.

*Klebsiella* bacteria are known to form biofilms, which increases their capacity for virulence. KMI8 was shown to be capable of degrading *K*. *michiganensis* mono biofilms, and this is the first report of bacteriophages degrading biofilms of this host. It is known that bacteria, including *Klebsiella* spp, can develop bacteriophage resistance via structural barriers such as capsular polysaccharides [51], and that capsule degrading depolymerase enzymes are important in allowing bacteriophages to break down *K*. *pneumoniae* biofilms [52]. The *K*. *michiganensis* strain KLEB011 which was used for the biofilm studies in our work, possessed the polysaccharide capsule KL70 locus, which is also found in *K*. *pneumoniae* [35, 41]. The genome of KMI8 possessed a putative endosialidase gene element (according to the Pfam database), embedded in an ORF which bears similarity to tail fibre elements (ORF 25). Endosialidases act as depolymerases, and are known to degrade the bacterial capsule polysaccharide polysialic acid [53–55]. The capacity to degrade *K*. *michiganensis* biofilms is potentially of clinical importance. Gut microbial dysbiosis is known to contribute to carcinogenesis, and while organisms such as *Fusobacterium nucleatum* have been shown to promote cancer progression by forming biofilms around tumours [56–58], *K*. *michiganensis* has not been directly implicated. However, extensive studies on the pathology caused by *K*. *michiganensis* are limited. Those that have been performed show that it is important clinically, as a nosocomial pathogen able to cause blood borne infection in immunocompromised patients, and severe, possibly fatal gut damage in preterm infants [8–11]. Apart from their incapacity to penetrate bacterial biofilms [20], antibiotics may increase colonisation and biofilm formation by *Klebsiella* spp [18]. Recent reports of the purification of enzymes capable of interfering with quorum sensing (involved in cell-cell communication in bacterial biofilms) offer new prospects for disruption/prevention of biofilm formation [59]. The use of bacteriophages similar to KMI8 which are specific for *K*. *michiganensis* also offer potential, especially in specificity in rectifying dysbiosis in the gut and invasion of other tissues. To this end, bacteriophage cocktails have been shown to be more effective than single bacteriophages in disrupting biofilms *in vitro* as well as in prevention and elimination of biofilm related infections [21]. As such, further analysis of bacteriophages which lyse *K*. *michiganensis*, and their capacity to be applied in cocktails to disrupt biofilms, is warranted.

Finally, as mentioned above, extensive studies on the pathology caused by *K*. *michiganensis* are limited, and the differentiation between this species and *K*. *oxytoca* requires genomic analyses such as WGS. This has important implications for *Klebsiella* speciation in diagnostic laboratories, as such molecular techniques are not readily available in routine clinical laboratories. Therefore, the occurrence and significance of *K*. *michiganensis* in clinical disease is likely to have been under-estimated, and this newly identified species may contribute to diseases previously reported to be caused by *K*. *oxytoca* and other members of the genus. This also highlights the importance of WGS of *Klebsiella* spp. when considering establishment of bacteriophage banks for integration in clinical practice [60]. Given the tendency to misidentify *K*. *michiganensis* as *K*. *oxytoca* in clinical settings, bacteriophages against *K*. *michiganensis*, such as KMI8, may be considered for inclusion in bacteriophage cocktails for future treatment of clinically diagnosed *K*. *oxytoca* infections.

## Supporting information

S1 Table (DOCX)
