# Supplementary material for: Novel Drexlerviridae bacteriophage KMI8 with specific lytic activity against Klebsiella michiganensis and its biofilms
Source: PLoS One. 2021 Sep 7;16(9):e0257102. doi: 10.1371/journal.pone.0257102 (PMC8423285; doi:10.1371/journal.pone.0257102)
Supplement: S1 Table — (DOCX) [file pone.0257102.s001.docx]

|  | **KLEB001** | **KLEB002** | **KLEB006** | **KLEB007** | **KLEB009** | **KLEB011** | **KLEB012** | **KLEB013** | **KLEB014** | **KLEB015** | **KLEB016** |
| --- | --- | --- | --- | --- | --- | --- | --- | --- | --- | --- | --- |
| **KLEB001** | * | 98.83 | 98.62 | 98.92 | 98.74 | 83.02 | 82.52 | 82.87 | 82.98 | 83.23 | 83.18 |
|  |  | *(89.32)* | *(89.67)* | *(88.98)* | *(86.42)* | *(75.04)* | *(73.14)* | *(73.66)* | *(74.78)* | *(75.50)* | *(75.80)* |
| **KLEB002** | 98.63 | * | 98.50 | 98.73 | 98.65 | 82.81 | 82.25 | 82.68 | 82.86 | 83.27 | 82.99 |
|  | *(88.57)* |  | *(89.91)* | *(89.84)* | *(88.29)* | *(74.15)* | *(73.09)* | *(73.64)* | *(75.25)* | *(75.74)* | *(76.33)* |
| **KLEB006** | 98.76 | 98.83 | * | 98.93 | 98.87 | 82.95 | 82.33 | 82.72 | 82.69 | 82.92 | 82.75 |
|  | *(89.03)* | *(90.07)* |  | *(90.18)* | *(87.95)* | *(75.58)* | *(74.49)* | *(75.21)* | *(75.52)* | *(76.02)* | *(75.70)* |
| **KLEB007** | 98.74 | 98.92 | 98.78 | * | 98.76 | 82.68 | 82.34 | 82.71 | 82.60 | 82.69 | 82.65 |
|  | *(90.49)* | *(91.92)* | *(92.20)* |  | *(90.07)* | *(76.53)* | *(76.14)* | *(75.74)* | *(76.23)* | *(76.38)* | *(76.96)* |
| **KLEB009** | 98.75 | 98.86 | 98.81 | 98.89 | * | 82.73 | 82.32 | 82.67 | 82.62 | 82.68 | 82.63 |
|  | *(88.64)* | *(91.26)* | *(90.78)* | *(90.78)* |  | *(76.33)* | *(74.43)* | *(75.97)* | *(76.59)* | *(76.26)* | *(77.13)* |
| **KLEB011** | 82.67 | 82.65 | 82.59 | 82.48 | 82.47 | * | 91.79 | 98.71 | 98.29 | 98.93 | 98.75 |
|  | *(67.49)* | *(67.40)* | *(68.65)* | *(67.56)* | *(67.03)* |  | *(79.36)* | *(87.62)* | *(87.31)* | *(89.90)* | *(87.48)* |
| **KLEB012** | 82.30 | 82.23 | 82.17 | 82.30 | 82.15 | 91.94 | * | 91.95 | 91.84 | 92.00 | 91.89 |
|  | *(71.57)* | *(71.98)* | *(73.10)* | *(72.93)* | *(71.14)* | *(85.92)* |  | *(86.56)* | *(85.67)* | *(86.48)* | *(86.71)* |
| **KLEB013** | 82.41 | 82.40 | 82.37 | 82.41 | 82.37 | 98.81 | 91.78 | * | 98.64 | 98.98 | 99.16 |
|  | *(69.10)* | *(69.72)* | *(71.05)* | *(69.93)* | *(69.67)* | *(91.29)* | *(83.50)* |  | *(92.23)* | *(91.86)* | *(94.40)* |
| **KLEB014** | 82.51 | 82.60 | 82.37 | 82.41 | 82.34 | 98.12 | 91.55 | 98.46 | * | 98.13 | 98.16 |
|  | *(66.96)* | *(68.07)* | *(68.09)* | *(67.15)* | *(66.97)* | *(87.05)* | *(78.71)* | *(88.02)* |  | *(88.72)* | *(89.35)* |
| **KLEB015** | 82.79 | 82.94 | 82.51 | 82.43 | 82.35 | 98.63 | 91.60 | 98.71 | 98.12 | * | 98.46 |
|  | *(67.05)* | *(68.07)* | *(68.01)* | *(66.54)* | *(66.18)* | *(89.20)* | *(79.13)* | *(87.16)* | *(87.97)* |  | *(88.95)* |
| **KLEB016** | 82.62 | 82.60 | 82.16 | 82.23 | 82.20 | 98.46 | 91.30 | 98.82 | 98.00 | 98.39 | * |
|  | *(65.13)* | *(66.29)* | *(65.65)* | *(64.95)* | *(64.77)* | *(82.98)* | *(76.32)* | *(85.91)* | *(85.25)* | *(85.29)* |  |

**S1 Table.**

Average Nucleotide identity for KLEB001, KLEB002, KLEB006, KLEB007, KLEB009 and KLEB011-016. Black = Average Nucleotide Identity (%); Red, italicised, in parentheses = % aligned nucleotides
